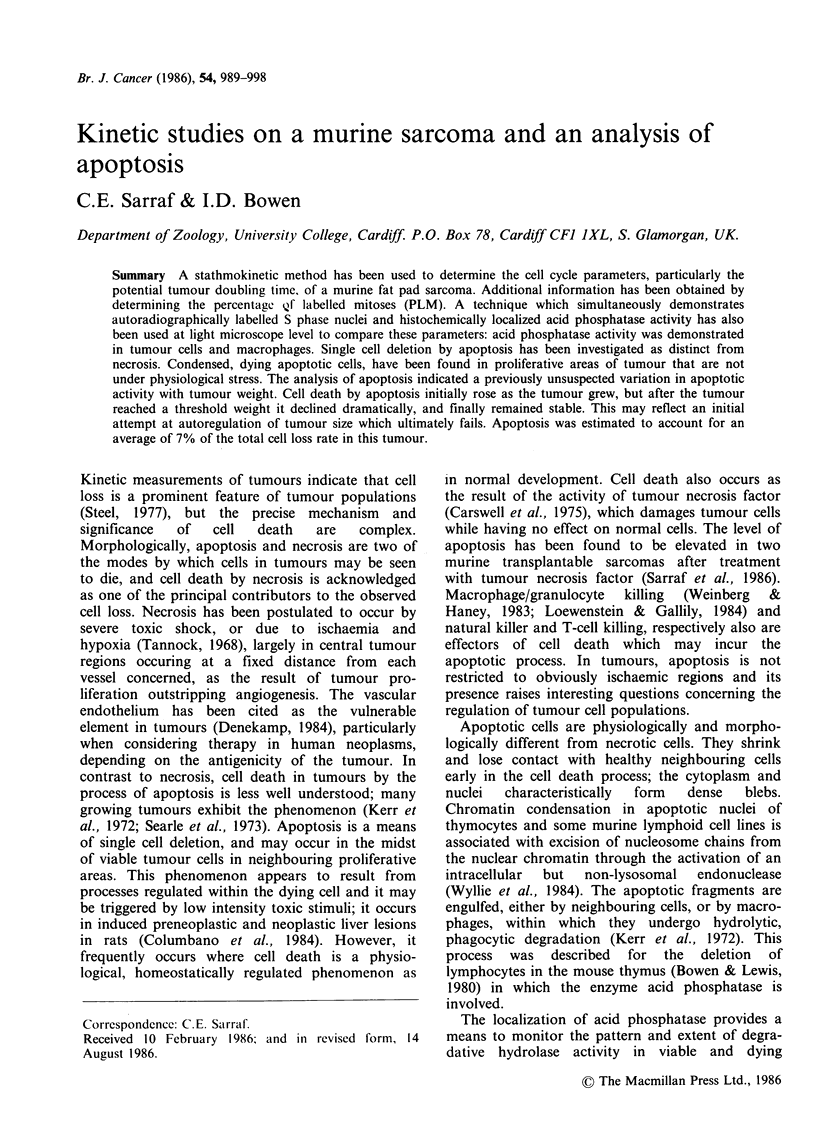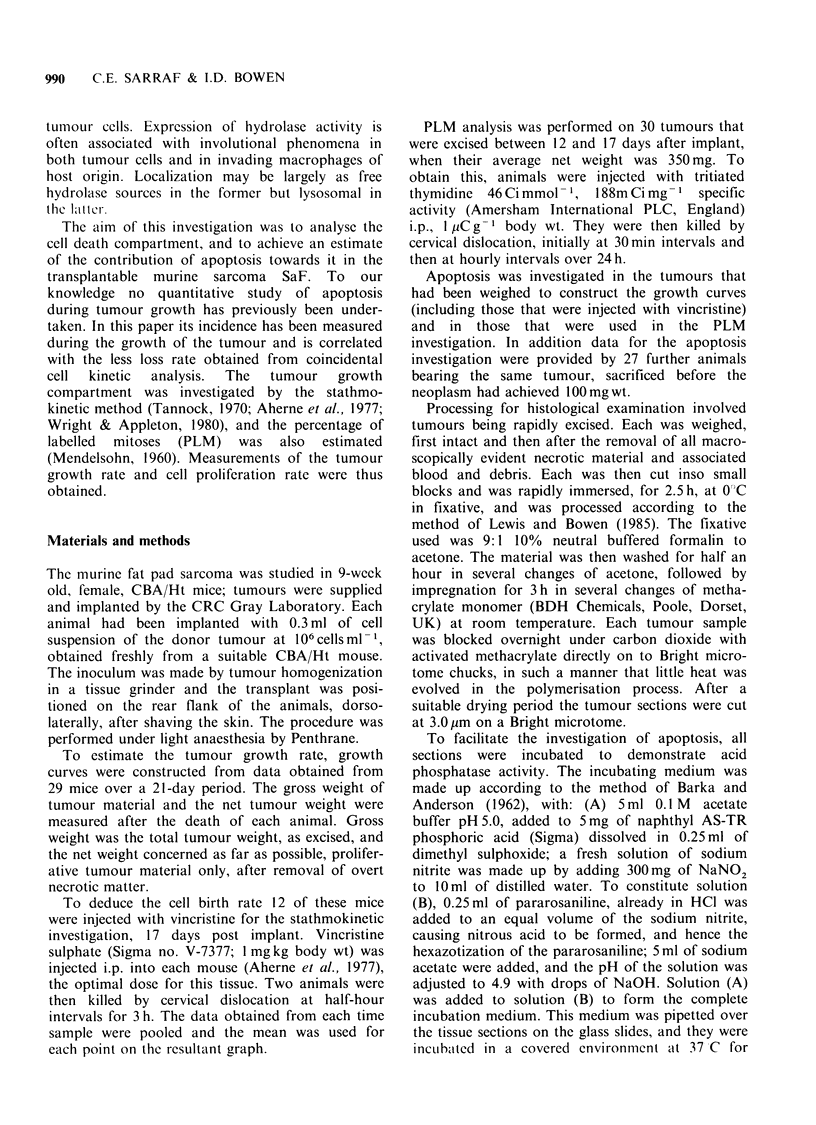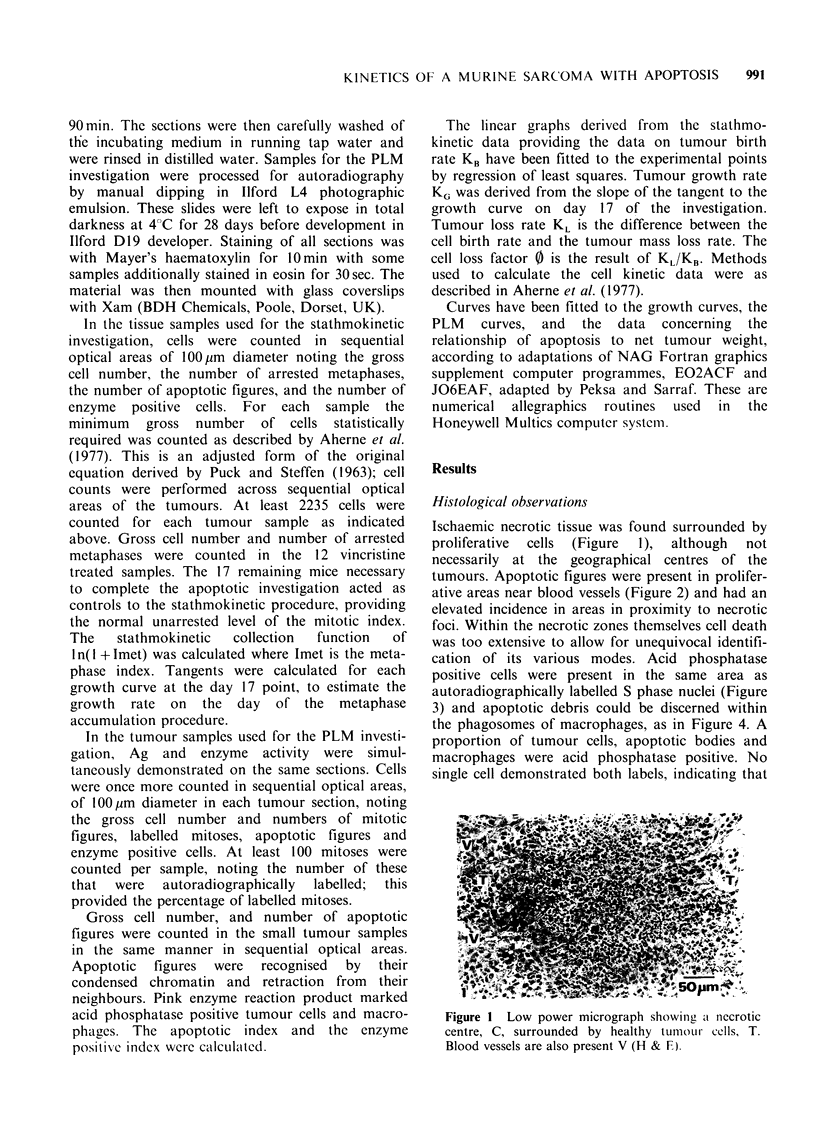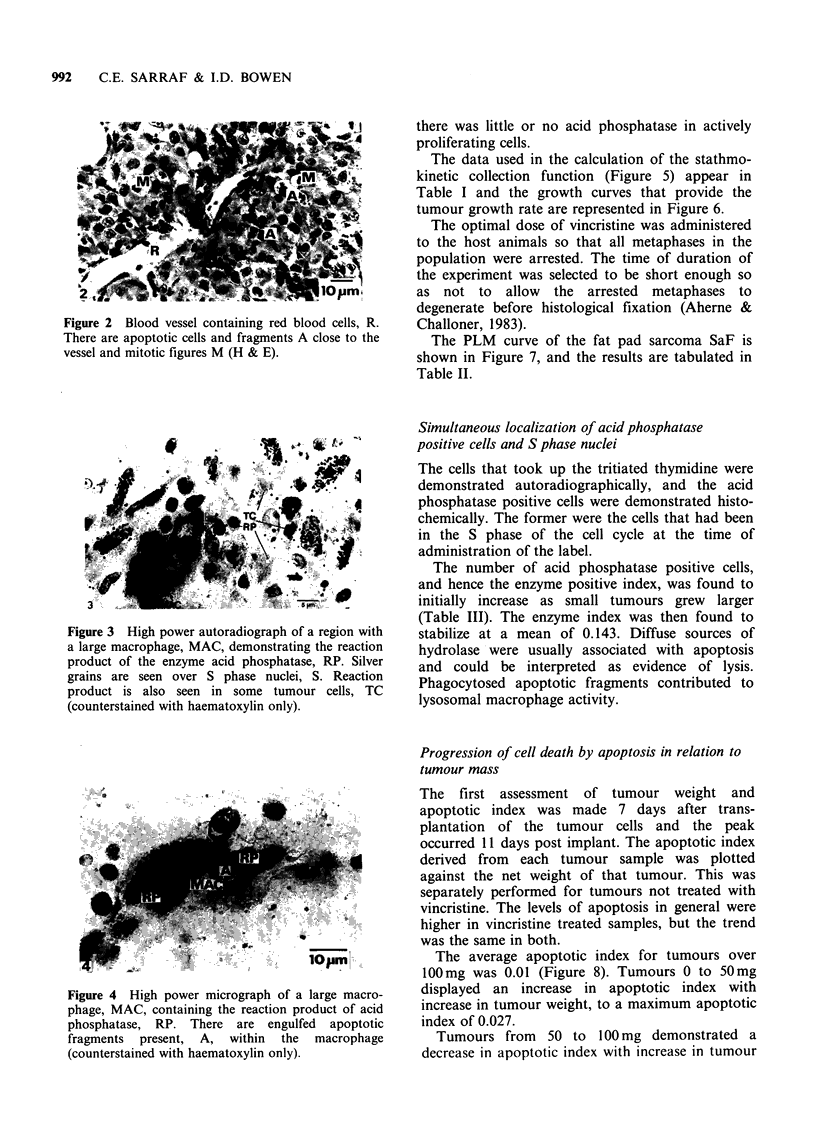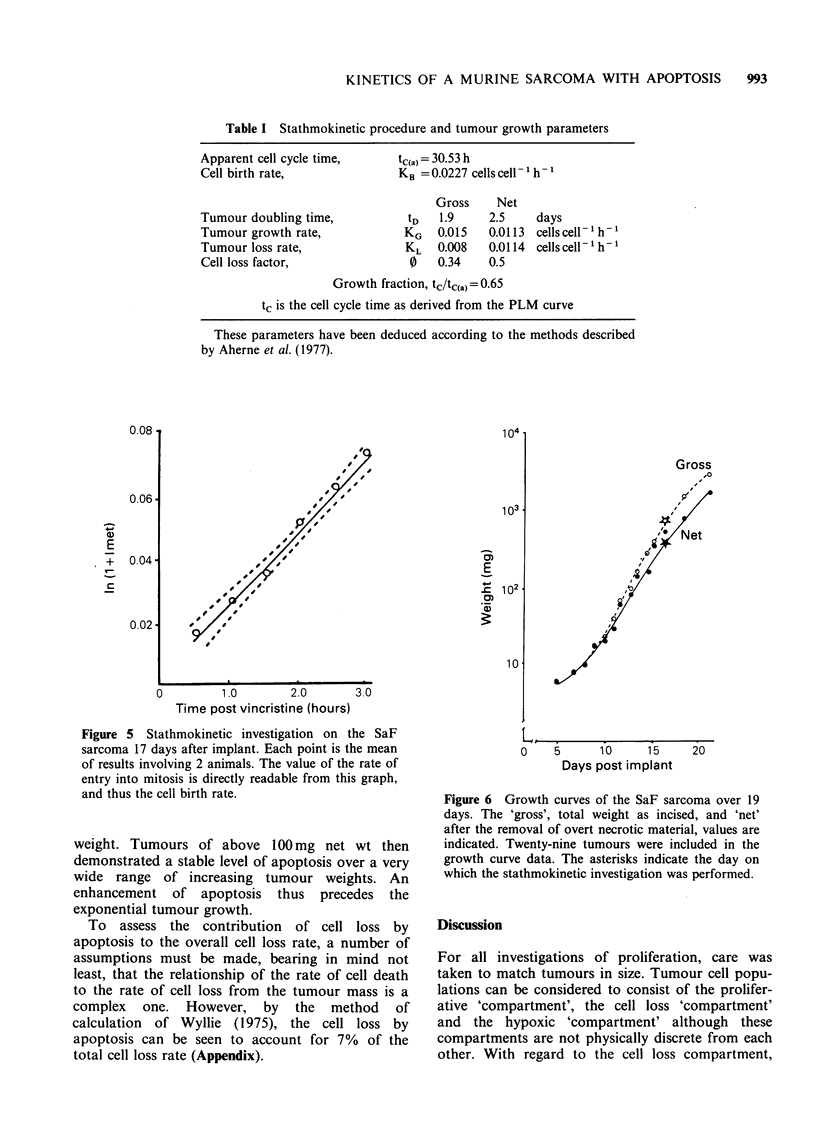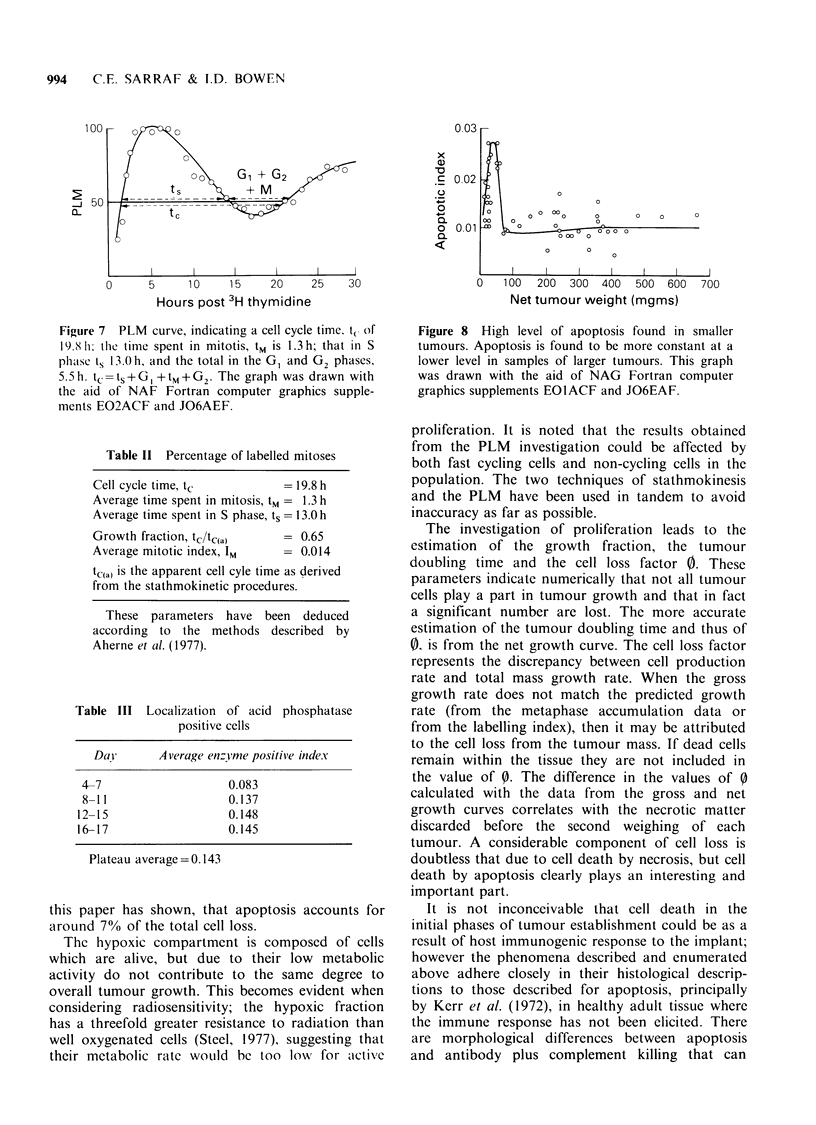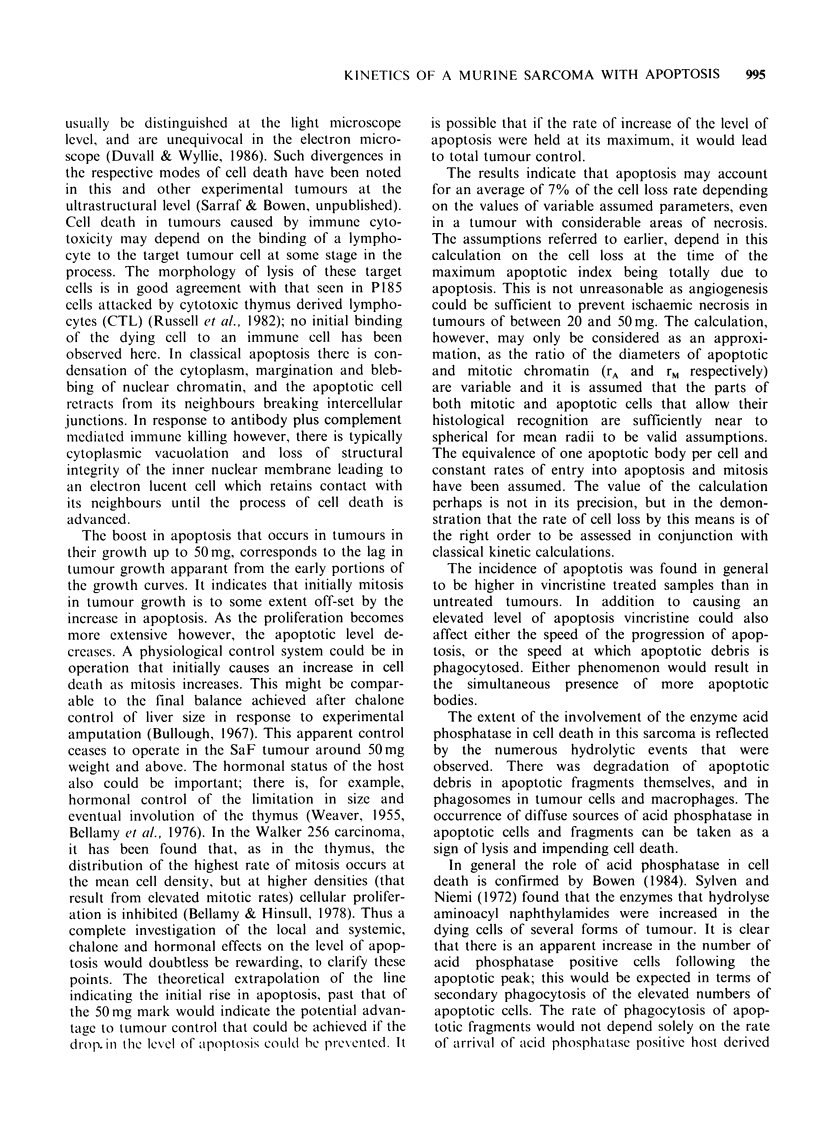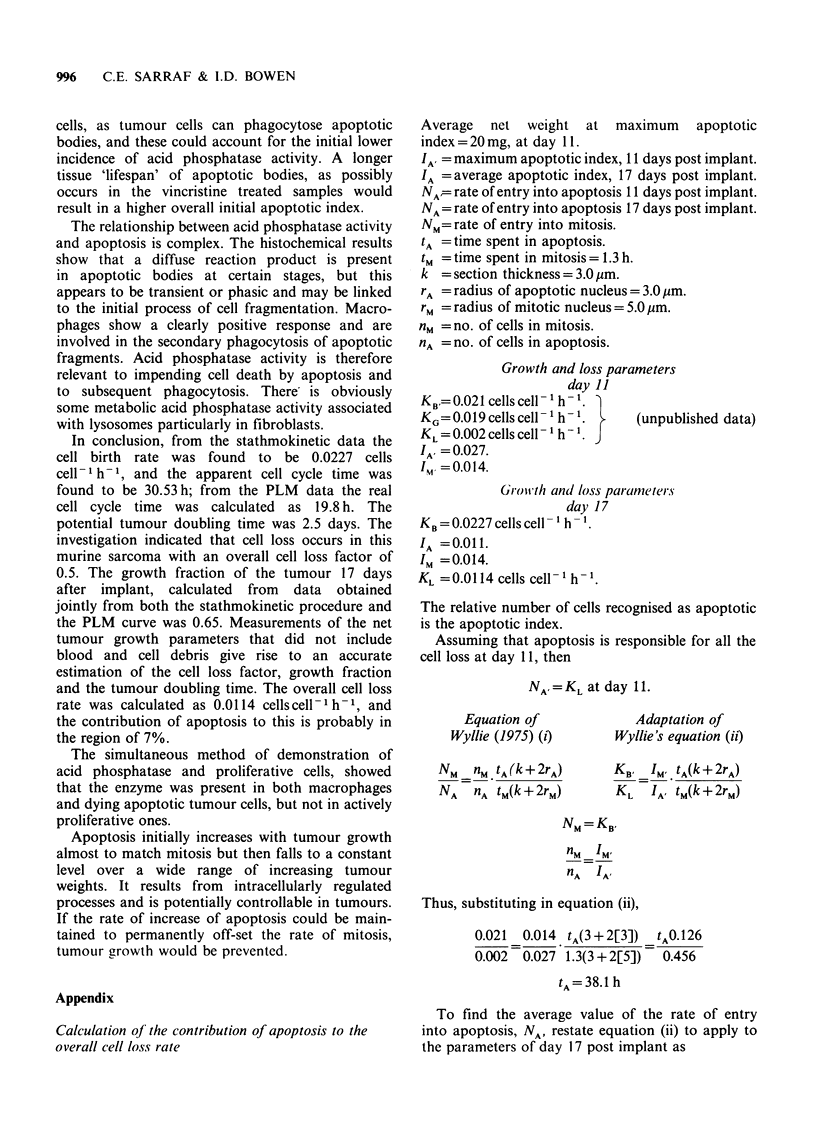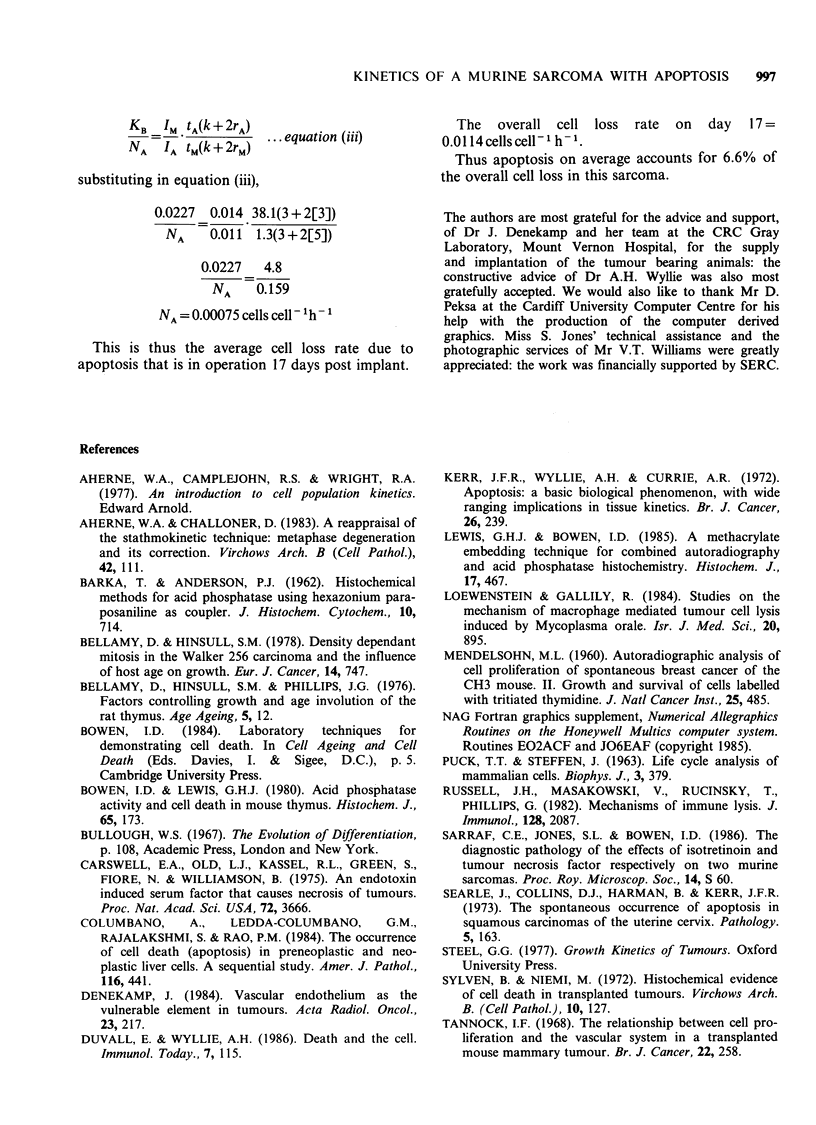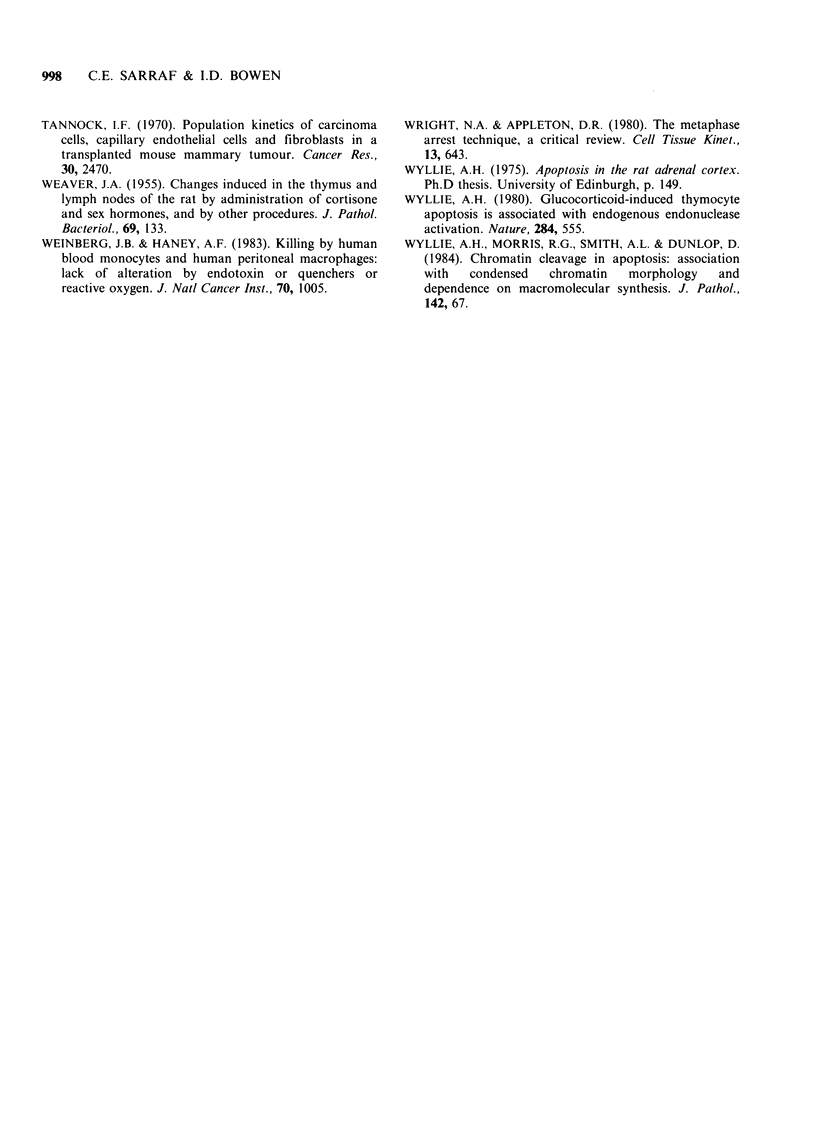# Kinetic studies on a murine sarcoma and an analysis of apoptosis.

**DOI:** 10.1038/bjc.1986.271

**Published:** 1986-12

**Authors:** C. E. Sarraf, I. D. Bowen

## Abstract

**Images:**


*Br. J. Cancer* (1986), **54**, 989–998

### Kinetic studies on a murine sarcoma and an analysis of apoptosis

C.E. Sarraf & I.D. Bowen

*Department of Zoology, University College, Cardiff. P.O. Box 78, Cardiff CF1 1XL, S. Glamorgan, UK.*

**Summary** A stathmokinetic method has been used to determine the cell cycle parameters, particularly the potential tumour doubling time, of a murine fat pad sarcoma. Additional information has been obtained by determining the percentage of labelled mitoses (PLM). A technique which simultaneously demonstrates autoradiographically labelled S phase nuclei and histochemically localized acid phosphatase activity has also been used at light microscope level to compare these parameters: acid phosphatase activity was demonstrated in tumour cells and macrophages. Single cell deletion by apoptosis has been investigated as distinct from necrosis. Condensed, dying apoptotic cells, have been found in proliferative areas of tumour that are not under physiological stress. The analysis of apoptosis indicated a previously unsuspected variation in apoptotic activity with tumour weight. Cell death by apoptosis initially rose as the tumour grew, but after the tumour reached a threshold weight it declined dramatically, and finally remained stable. This may reflect an initial attempt at autoregulation of tumour size which ultimately fails. Apoptosis was estimated to account for an average of 7% of the total cell loss rate in this tumour.

Kinetic measurements of tumours indicate that cell loss is a prominent feature of tumour populations (Steel, 1977), but the precise mechanism and significance of cell death are complex. Morphologically, apoptosis and necrosis are two of the modes by which cells in tumours may be seen to die, and cell death by necrosis is acknowledged as one of the principal contributors to the observed cell loss. Necrosis has been postulated to occur by severe toxic shock, or due to ischaemia and hypoxia (Tannock, 1968), largely in central tumour regions occuring at a fixed distance from each vessel concerned, as the result of tumour proliferation outstripping angiogenesis. The vascular endothelium has been cited as the vulnerable element in tumours (Denekamp, 1984), particularly when considering therapy in human neoplasms, depending on the antigenicity of the tumour. In contrast to necrosis, cell death in tumours by the process of apoptosis is less well understood; many growing tumours exhibit the phenomenon (Kerr *et al.*, 1972; Searle *et al.*, 1973). Apoptosis is a means of single cell deletion, and may occur in the midst of viable tumour cells in neighbouring proliferative areas. This phenomenon appears to result from processes regulated within the dying cell and it may be triggered by low intensity toxic stimuli; it occurs in induced preneoplastic and neoplastic liver lesions in rats (Columbano *et al.*, 1984). However, it frequently occurs where cell death is a physiological, homeostatically regulated phenomenon as in normal development. Cell death also occurs as the result of the activity of tumour necrosis factor (Carswell *et al.*, 1975), which damages tumour cells while having no effect on normal cells. The level of apoptosis has been found to be elevated in two murine transplantable sarcomas after treatment with tumour necrosis factor (Sarraf *et al.*, 1986). Macrophage/granulocyte killing (Weinberg & Haney, 1983; Loewenstein & Gallily, 1984) and natural killer and T-cell killing, respectively also are effectors of cell death which may incur the apoptotic process. In tumours, apoptosis is not restricted to obviously ischaemic regions and its presence raises interesting questions concerning the regulation of tumour cell populations.

Apoptotic cells are physiologically and morphologically different from necrotic cells. They shrink and lose contact with healthy neighbouring cells early in the cell death process; the cytoplasm and nuclei characteristically form dense blebs. Chromatin condensation in apoptotic nuclei of thymocytes and some murine lymphoid cell lines is associated with excision of nucleosome chains from the nuclear chromatin through the activation of an intracellular but non-lysosomal endonuclease (Wyllie *et al.*, 1984). The apoptotic fragments are engulfed, either by neighbouring cells, or by macrophages, within which they undergo hydrolytic, phagocytic degradation (Kerr *et al.*, 1972). This process was described for the deletion of lymphocytes in the mouse thymus (Bowen & Lewis, 1980) in which the enzyme acid phosphatase is involved.

The localization of acid phosphatase provides a means to monitor the pattern and extent of degradative hydrolase activity in viable and dying

Correspondence: C.E. Sarraf.
Received 10 February 1986; and in revised form, 14 August 1986.

© The Macmillan Press Ltd., 1986

tumour cells. Expression of hydrolase activity is often associated with involutional phenomena in both tumour cells and in invading macrophages of host origin. Localization may be largely as free hydrolase sources in the former but lysosomal in the latter.

The aim of this investigation was to analyse the cell death compartment, and to achieve an estimate of the contribution of apoptosis towards it in the transplantable murine sarcoma SaF. To our knowledge no quantitative study of apoptosis during tumour growth has previously been undertaken. In this paper its incidence has been measured during the growth of the tumour and is correlated with the less loss rate obtained from coincidental cell kinetic analysis. The tumour growth compartment was investigated by the stathmokinetic method (Tannock, 1970; Aherne *et al.*, 1977; Wright & Appleton, 1980), and the percentage of labelled mitoses (PLM) was also estimated (Mendelsohn, 1960). Measurements of the tumour growth rate and cell proliferation rate were thus obtained.

### Materials and methods

The murine fat pad sarcoma was studied in 9-week old, female, CBA/Ht mice; tumours were supplied and implanted by the CRC Gray Laboratory. Each animal had been implanted with 0.3 ml of cell suspension of the donor tumour at $10^6$ cells $ml^{-1}$, obtained freshly from a suitable CBA/Ht mouse. The inoculum was made by tumour homogenization in a tissue grinder and the transplant was positioned on the rear flank of the animals, dorsolaterally, after shaving the skin. The procedure was performed under light anaesthesia by Penthrane.

To estimate the tumour growth rate, growth curves were constructed from data obtained from 29 mice over a 21-day period. The gross weight of tumour material and the net tumour weight were measured after the death of each animal. Gross weight was the total tumour weight, as excised, and the net weight concerned as far as possible, proliferative tumour material only, after removal of overt necrotic matter.

To deduce the cell birth rate 12 of these mice were injected with vincristine for the stathmokinetic investigation, 17 days post implant. Vincristine sulphate (Sigma no. V-7377; 1 mg kg body wt) was injected i.p. into each mouse (Aherne *et al.*, 1977), the optimal dose for this tissue. Two animals were then killed by cervical dislocation at half-hour intervals for 3 h. The data obtained from each time sample were pooled and the mean was used for each point on the resultant graph.

PLM analysis was performed on 30 tumours that were excised between 12 and 17 days after implant, when their average net weight was 350 mg. To obtain this, animals were injected with tritiated thymidine 46 Ci $mmol^{-1}$, 188m Ci $mg^{-1}$ specific activity (Amersham International PLC, England) i.p., 1 $\mu$C $g^{-1}$ body wt. They were then killed by cervical dislocation, initially at 30 min intervals and then at hourly intervals over 24 h.

Apoptosis was investigated in the tumours that had been weighed to construct the growth curves (including those that were injected with vincristine) and in those that were used in the PLM investigation. In addition data for the apoptosis investigation were provided by 27 further animals bearing the same tumour, sacrificed before the neoplasm had achieved 100 mg wt.

Processing for histological examination involved tumours being rapidly excised. Each was weighed, first intact and then after the removal of all macroscopically evident necrotic material and associated blood and debris. Each was then cut inso small blocks and was rapidly immersed, for 2.5 h, at 0°C in fixative, and was processed according to the method of Lewis and Bowen (1985). The fixative used was 9:1 10% neutral buffered formalin to acetone. The material was then washed for half an hour in several changes of acetone, followed by impregnation for 3 h in several changes of methacrylate monomer (BDH Chemicals, Poole, Dorset, UK) at room temperature. Each tumour sample was blocked overnight under carbon dioxide with activated methacrylate directly on to Bright microtome chucks, in such a manner that little heat was evolved in the polymerisation process. After a suitable drying period the tumour sections were cut at 3.0 $\mu$m on a Bright microtome.

To facilitate the investigation of apoptosis, all sections were incubated to demonstrate acid phosphatase activity. The incubating medium was made up according to the method of Barka and Anderson (1962), with: (A) 5 ml 0.1 M acetate buffer pH 5.0, added to 5 mg of naphthyl AS-TR phosphoric acid (Sigma) dissolved in 0.25 ml of dimethyl sulphoxide; a fresh solution of sodium nitrite was made up by adding 300 mg of $NaNO_2$ to 10 ml of distilled water. To constitute solution (B), 0.25 ml of pararosaniline, already in HCl was added to an equal volume of the sodium nitrite, causing nitrous acid to be formed, and hence the hexazotization of the pararosaniline; 5 ml of sodium acetate were added, and the pH of the solution was adjusted to 4.9 with drops of NaOH. Solution (A) was added to solution (B) to form the complete incubation medium. This medium was pipetted over the tissue sections on the glass slides, and they were incubated in a covered environment at 37°C for

90 min. The sections were then carefully washed of the incubating medium in running tap water and were rinsed in distilled water. Samples for the PLM investigation were processed for autoradiography by manual dipping in Ilford L4 photographic emulsion. These slides were left to expose in total darkness at 4°C for 28 days before development in Ilford D19 developer. Staining of all sections was with Mayer's haematoxylin for 10 min with some samples additionally stained in eosin for 30 sec. The material was then mounted with glass coverslips with Xam (BDH Chemicals, Poole, Dorset, UK).

In the tissue samples used for the stathmokinetic investigation, cells were counted in sequential optical areas of 100 $\mu$m diameter noting the gross cell number, the number of arrested metaphases, the number of apoptotic figures, and the number of enzyme positive cells. For each sample the minimum gross number of cells statistically required was counted as described by Aherne *et al.* (1977). This is an adjusted form of the original equation derived by Puck and Steffen (1963); cell counts were performed across sequential optical areas of the tumours. At least 2235 cells were counted for each tumour sample as indicated above. Gross cell number and number of arrested metaphases were counted in the 12 vincristine treated samples. The 17 remaining mice necessary to complete the apoptotic investigation acted as controls to the stathmokinetic procedure, providing the normal unarrested level of the mitotic index. The stathmokinetic collection function of $\ln(1 + \text{Imet})$ was calculated where Imet is the metaphase index. Tangents were calculated for each growth curve at the day 17 point, to estimate the growth rate on the day of the metaphase accumulation procedure.

In the tumour samples used for the PLM investigation, Ag and enzyme activity were simultaneously demonstrated on the same sections. Cells were once more counted in sequential optical areas, of 100 $\mu$m diameter in each tumour section, noting the gross cell number and numbers of mitotic figures, labelled mitoses, apoptotic figures and enzyme positive cells. At least 100 mitoses were counted per sample, noting the number of these that were autoradiographically labelled; this provided the percentage of labelled mitoses.

Gross cell number, and number of apoptotic figures were counted in the small tumour samples in the same manner in sequential optical areas. Apoptotic figures were recognised by their condensed chromatin and retraction from their neighbours. Pink enzyme reaction product marked acid phosphatase positive tumour cells and macrophages. The apoptotic index and the enzyme positive index were calculated.

The linear graphs derived from the stathmokinetic data providing the data on tumour birth rate $K_B$ have been fitted to the experimental points by regression of least squares. Tumour growth rate $K_G$ was derived from the slope of the tangent to the growth curve on day 17 of the investigation. Tumour loss rate $K_L$ is the difference between the cell birth rate and the tumour mass loss rate. The cell loss factor $\emptyset$ is the result of $K_L/K_B$. Methods used to calculate the cell kinetic data were as described in Aherne *et al.* (1977).

Curves have been fitted to the growth curves, the PLM curves, and the data concerning the relationship of apoptosis to net tumour weight, according to adaptations of NAG Fortran graphics supplement computer programmes, EO2ACF and JO6EAF, adapted by Peksa and Sarraf. These are numerical allegraphics routines used in the Honeywell Multics computer system.

### Results

### *Histological observations*

Ischaemic necrotic tissue was found surrounded by proliferative cells (Figure 1), although not necessarily at the geographical centres of the tumours. Apoptotic figures were present in proliferative areas near blood vessels (Figure 2) and had an elevated incidence in areas in proximity to necrotic foci. Within the necrotic zones themselves cell death was too extensive to allow for unequivocal identification of its various modes. Acid phosphatase positive cells were present in the same area as autoradiographically labelled S phase nuclei (Figure 3) and apoptotic debris could be discerned within the phagosomes of macrophages, as in Figure 4. A proportion of tumour cells, apoptotic bodies and macrophages were acid phosphatase positive. No single cell demonstrated both labels, indicating that

**Figure 1** Low power micrograph showing a necrotic centre, C, surrounded by healthy tumour cells, T. Blood vessels are also present V (H & E).

**Figure 2** Blood vessel containing red blood cells, R. There are apoptotic cells and fragments A close to the vessel and mitotic figures M (H & E).

**Figure 3** High power autoradiograph of a region with a large macrophage, MAC, demonstrating the reaction product of the enzyme acid phosphatase, RP. Silver grains are seen over S phase nuclei, S. Reaction product is also seen in some tumour cells, TC (counterstained with haematoxylin only).

**Figure 4** High power micrograph of a large macrophage, MAC, containing the reaction product of acid phosphatase, RP. There are engulfed apoptotic fragments present, A, within the macrophage (counterstained with haematoxylin only).

there was little or no acid phosphatase in actively proliferating cells.

The data used in the calculation of the stathmokinetic collection function (Figure 5) appear in Table I and the growth curves that provide the tumour growth rate are represented in Figure 6.

The optimal dose of vincristine was administered to the host animals so that all metaphases in the population were arrested. The time of duration of the experiment was selected to be short enough so as not to allow the arrested metaphases to degenerate before histological fixation (Aherne & Challoner, 1983).

The PLM curve of the fat pad sarcoma SaF is shown in Figure 7, and the results are tabulated in Table II.

*Simultaneous localization of acid phosphatase positive cells and S phase nuclei*

The cells that took up the tritiated thymidine were demonstrated autoradiographically, and the acid phosphatase positive cells were demonstrated histochemically. The former were the cells that had been in the S phase of the cell cycle at the time of administration of the label.

The number of acid phosphatase positive cells, and hence the enzyme positive index, was found to initially increase as small tumours grew larger (Table III). The enzyme index was then found to stabilize at a mean of 0.143. Diffuse sources of hydrolase were usually associated with apoptosis and could be interpreted as evidence of lysis. Phagocytosed apoptotic fragments contributed to lysosomal macrophage activity.

*Progression of cell death by apoptosis in relation to tumour mass*

The first assessment of tumour weight and apoptotic index was made 7 days after transplantation of the tumour cells and the peak occurred 11 days post implant. The apoptotic index derived from each tumour sample was plotted against the net weight of that tumour. This was separately performed for tumours not treated with vincristine. The levels of apoptosis in general were higher in vincristine treated samples, but the trend was the same in both.

The average apoptotic index for tumours over 100 mg was 0.01 (Figure 8). Tumours 0 to 50 mg displayed an increase in apoptotic index with increase in tumour weight, to a maximum apoptotic index of 0.027.

Tumours from 50 to 100 mg demonstrated a decrease in apoptotic index with increase in tumour

**Table I** Stathmokinetic procedure and tumour growth parameters

| | | | | |
|---|---|---|---|---|
| Apparent cell cycle time, | $t_{C(a)} = 30.53$ h | | | |
| Cell birth rate, | $K_B = 0.0227$ cells cell$^{-1}$ h$^{-1}$ | | | |
| | | Gross | Net | |
| Tumour doubling time, | $t_D$ | 1.9 | 2.5 | days |
| Tumour growth rate, | $K_G$ | 0.015 | 0.0113 | cells cell$^{-1}$ h$^{-1}$ |
| Tumour loss rate, | $K_L$ | 0.008 | 0.0114 | cells cell$^{-1}$ h$^{-1}$ |
| Cell loss factor, | $\phi$ | 0.34 | 0.5 | |
| Growth fraction, $t_C/t_{C(a)} = 0.65$ | | | | |
| $t_C$ is the cell cycle time as derived from the PLM curve | | | | |

These parameters have been deduced according to the methods described by Aherne *et al.* (1977).

**Figure 5** Stathmokinetic investigation on the SaF sarcoma 17 days after implant. Each point is the mean of results involving 2 animals. The value of the rate of entry into mitosis is directly readable from this graph, and thus the cell birth rate.

**Figure 6** Growth curves of the SaF sarcoma over 19 days. The 'gross', total weight as incised, and 'net' after the removal of overt necrotic material, values are indicated. Twenty-nine tumours were included in the growth curve data. The asterisks indicate the day on which the stathmokinetic investigation was performed.

weight. Tumours of above 100 mg net wt then demonstrated a stable level of apoptosis over a very wide range of increasing tumour weights. An enhancement of apoptosis thus precedes the exponential tumour growth.

To assess the contribution of cell loss by apoptosis to the overall cell loss rate, a number of assumptions must be made, bearing in mind not least, that the relationship of the rate of cell death to the rate of cell loss from the tumour mass is a complex one. However, by the method of calculation of Wyllie (1975), the cell loss by apoptosis can be seen to account for 7% of the total cell loss rate (**Appendix**).

### Discussion

For all investigations of proliferation, care was taken to match tumours in size. Tumour cell populations can be considered to consist of the proliferative 'compartment', the cell loss 'compartment' and the hypoxic 'compartment' although these compartments are not physically discrete from each other. With regard to the cell loss compartment,

**Figure 7** PLM curve, indicating a cell cycle time, $t_C$ of 19.8 h; the time spent in mitotis, $t_M$ is 1.3 h; that in S phase $t_S$ 13.0 h, and the total in the $G_1$ and $G_2$ phases, 5.5 h. $t_C = t_S + G_1 + t_M + G_2$. The graph was drawn with the aid of NAF Fortran computer graphics supplements EO2ACF and JO6AEF.

**Figure 8** High level of apoptosis found in smaller tumours. Apoptosis is found to be more constant at a lower level in samples of larger tumours. This graph was drawn with the aid of NAG Fortran computer graphics supplements EO1ACF and JO6EAF.

**Table II** Percentage of labelled mitoses

| | |
|---|---|
| Cell cycle time, $t_C$ | = 19.8 h |
| Average time spent in mitosis, $t_M$ | = 1.3 h |
| Average time spent in S phase, $t_S$ | = 13.0 h |
| Growth fraction, $t_C/t_{C(a)}$ | = 0.65 |
| Average mitotic index, $I_M$ | = 0.014 |

$t_{C(a)}$ is the apparent cell cyle time as derived from the stathmokinetic procedures.

These parameters have been deduced according to the methods described by Aherne *et al.* (1977).

**Table III** Localization of acid phosphatase positive cells

| *Day* | *Average enzyme positive index* |
|---|---|
| 4–7 | 0.083 |
| 8–11 | 0.137 |
| 12–15 | 0.148 |
| 16–17 | 0.145 |

Plateau average = 0.143

this paper has shown, that apoptosis accounts for around 7% of the total cell loss.

The hypoxic compartment is composed of cells which are alive, but due to their low metabolic activity do not contribute to the same degree to overall tumour growth. This becomes evident when considering radiosensitivity; the hypoxic fraction has a threefold greater resistance to radiation than well oxygenated cells (Steel, 1977), suggesting that their metabolic rate would be too low for active proliferation. It is noted that the results obtained from the PLM investigation could be affected by both fast cycling cells and non-cycling cells in the population. The two techniques of stathmokinesis and the PLM have been used in tandem to avoid inaccuracy as far as possible.

The investigation of proliferation leads to the estimation of the growth fraction, the tumour doubling time and the cell loss factor $\emptyset$. These parameters indicate numerically that not all tumour cells play a part in tumour growth and that in fact a significant number are lost. The more accurate estimation of the tumour doubling time and thus of $\emptyset$, is from the net growth curve. The cell loss factor represents the discrepancy between cell production rate and total mass growth rate. When the gross growth rate does not match the predicted growth rate (from the metaphase accumulation data or from the labelling index), then it may be attributed to the cell loss from the tumour mass. If dead cells remain within the tissue they are not included in the value of $\emptyset$. The difference in the values of $\emptyset$ calculated with the data from the gross and net growth curves correlates with the necrotic matter discarded before the second weighing of each tumour. A considerable component of cell loss is doubtless that due to cell death by necrosis, but cell death by apoptosis clearly plays an interesting and important part.

It is not inconceivable that cell death in the initial phases of tumour establishment could be as a result of host immunogenic response to the implant; however the phenomena described and enumerated above adhere closely in their histological descriptions to those described for apoptosis, principally by Kerr *et al.* (1972), in healthy adult tissue where the immune response has not been elicited. There are morphological differences between apoptosis and antibody plus complement killing that can usually be distinguished at the light microscope level, and are unequivocal in the electron microscope (Duvall & Wyllie, 1986). Such divergences in the respective modes of cell death have been noted in this and other experimental tumours at the ultrastructural level (Sarraf & Bowen, unpublished). Cell death in tumours caused by immune cytotoxicity may depend on the binding of a lymphocyte to the target tumour cell at some stage in the process. The morphology of lysis of these target cells is in good agreement with that seen in P185 cells attacked by cytotoxic thymus derived lymphocytes (CTL) (Russell *et al.*, 1982); no initial binding of the dying cell to an immune cell has been observed here. In classical apoptosis there is condensation of the cytoplasm, margination and blebbing of nuclear chromatin, and the apoptotic cell retracts from its neighbours breaking intercellular junctions. In response to antibody plus complement mediated immune killing however, there is typically cytoplasmic vacuolation and loss of structural integrity of the inner nuclear membrane leading to an electron lucent cell which retains contact with its neighbours until the process of cell death is advanced.

The boost in apoptosis that occurs in tumours in their growth up to 50 mg, corresponds to the lag in tumour growth apparant from the early portions of the growth curves. It indicates that initially mitosis in tumour growth is to some extent off-set by the increase in apoptosis. As the proliferation becomes more extensive however, the apoptotic level decreases. A physiological control system could be in operation that initially causes an increase in cell death as mitosis increases. This might be comparable to the final balance achieved after chalone control of liver size in response to experimental amputation (Bullough, 1967). This apparent control ceases to operate in the SaF tumour around 50 mg weight and above. The hormonal status of the host also could be important; there is, for example, hormonal control of the limitation in size and eventual involution of the thymus (Weaver, 1955, Bellamy *et al.*, 1976). In the Walker 256 carcinoma, it has been found that, as in the thymus, the distribution of the highest rate of mitosis occurs at the mean cell density, but at higher densities (that result from elevated mitotic rates) cellular proliferation is inhibited (Bellamy & Hinsull, 1978). Thus a complete investigation of the local and systemic, chalone and hormonal effects on the level of apoptosis would doubtless be rewarding, to clarify these points. The theoretical extrapolation of the line indicating the initial rise in apoptosis, past that of the 50 mg mark would indicate the potential advantage to tumour control that could be achieved if the drop in the level of apoptosis could be prevented. It is possible that if the rate of increase of the level of apoptosis were held at its maximum, it would lead to total tumour control.

The results indicate that apoptosis may account for an average of 7% of the cell loss rate depending on the values of variable assumed parameters, even in a tumour with considerable areas of necrosis. The assumptions referred to earlier, depend in this calculation on the cell loss at the time of the maximum apoptotic index being totally due to apoptosis. This is not unreasonable as angiogenesis could be sufficient to prevent ischaemic necrosis in tumours of between 20 and 50 mg. The calculation, however, may only be considered as an approximation, as the ratio of the diameters of apoptotic and mitotic chromatin ($r_A$ and $r_M$ respectively) are variable and it is assumed that the parts of both mitotic and apoptotic cells that allow their histological recognition are sufficiently near to spherical for mean radii to be valid assumptions. The equivalence of one apoptotic body per cell and constant rates of entry into apoptosis and mitosis have been assumed. The value of the calculation perhaps is not in its precision, but in the demonstration that the rate of cell loss by this means is of the right order to be assessed in conjunction with classical kinetic calculations.

The incidence of apoptotis was found in general to be higher in vincristine treated samples than in untreated tumours. In addition to causing an elevated level of apoptosis vincristine could also affect either the speed of the progression of apoptosis, or the speed at which apoptotic debris is phagocytosed. Either phenomenon would result in the simultaneous presence of more apoptotic bodies.

The extent of the involvement of the enzyme acid phosphatase in cell death in this sarcoma is reflected by the numerous hydrolytic events that were observed. There was degradation of apoptotic debris in apoptotic fragments themselves, and in phagosomes in tumour cells and macrophages. The occurrence of diffuse sources of acid phosphatase in apoptotic cells and fragments can be taken as a sign of lysis and impending cell death.

In general the role of acid phosphatase in cell death is confirmed by Bowen (1984). Sylven and Niemi (1972) found that the enzymes that hydrolyse aminoacyl naphthylamides were increased in the dying cells of several forms of tumour. It is clear that there is an apparent increase in the number of acid phosphatase positive cells following the apoptotic peak; this would be expected in terms of secondary phagocytosis of the elevated numbers of apoptotic cells. The rate of phagocytosis of apoptotic fragments would not depend solely on the rate of arrival of acid phosphatase positive host derived cells, as tumour cells can phagocytose apoptotic bodies, and these could account for the initial lower incidence of acid phosphatase activity. A longer tissue 'lifespan' of apoptotic bodies, as possibly occurs in the vincristine treated samples would result in a higher overall initial apoptotic index.

The relationship between acid phosphatase activity and apoptosis is complex. The histochemical results show that a diffuse reaction product is present in apoptotic bodies at certain stages, but this appears to be transient or phasic and may be linked to the initial process of cell fragmentation. Macrophages show a clearly positive response and are involved in the secondary phagocytosis of apoptotic fragments. Acid phosphatase activity is therefore relevant to impending cell death by apoptosis and to subsequent phagocytosis. There is obviously some metabolic acid phosphatase activity associated with lysosomes particularly in fibroblasts.

In conclusion, from the stathmokinetic data the cell birth rate was found to be 0.0227 cells $cell^{-1} h^{-1}$, and the apparent cell cycle time was found to be 30.53 h; from the PLM data the real cell cycle time was calculated as 19.8 h. The potential tumour doubling time was 2.5 days. The investigation indicated that cell loss occurs in this murine sarcoma with an overall cell loss factor of 0.5. The growth fraction of the tumour 17 days after implant, calculated from data obtained jointly from both the stathmokinetic procedure and the PLM curve was 0.65. Measurements of the net tumour growth parameters that did not include blood and cell debris give rise to an accurate estimation of the cell loss factor, growth fraction and the tumour doubling time. The overall cell loss rate was calculated as 0.0114 cells $cell^{-1} h^{-1}$, and the contribution of apoptosis to this is probably in the region of 7%.

The simultaneous method of demonstration of acid phosphatase and proliferative cells, showed that the enzyme was present in both macrophages and dying apoptotic tumour cells, but not in actively proliferative ones.

Apoptosis initially increases with tumour growth almost to match mitosis but then falls to a constant level over a wide range of increasing tumour weights. It results from intracellularly regulated processes and is potentially controllable in tumours. If the rate of increase of apoptosis could be maintained to permanently off-set the rate of mitosis, tumour growth would be prevented.

### Appendix

*Calculation of the contribution of apoptosis to the overall cell loss rate*

Average net weight at maximum apoptotic index = 20 mg, at day 11.

$I_{A'}$ = maximum apoptotic index, 11 days post implant.
$I_A$ = average apoptotic index, 17 days post implant.
$N_{A'}$ = rate of entry into apoptosis 11 days post implant.
$N_A$ = rate of entry into apoptosis 17 days post implant.
$N_M$ = rate of entry into mitosis.
$t_A$ = time spent in apoptosis.
$t_M$ = time spent in mitosis = 1.3 h.
$k$ = section thickness = 3.0 $\mu$m.
$r_A$ = radius of apoptotic nucleus = 3.0 $\mu$m.
$r_M$ = radius of mitotic nucleus = 5.0 $\mu$m.
$n_M$ = no. of cells in mitosis.
$n_A$ = no. of cells in apoptosis.

*Growth and loss parameters day 11*

$K_{B'} = 0.021$ cells $cell^{-1} h^{-1}$.

$K_G = 0.019$ cells $cell^{-1} h^{-1}$.  (unpublished data)

$K_L = 0.002$ cells $cell^{-1} h^{-1}$.

$I_{A'} = 0.027$.

$I_{M'} = 0.014$.

*Growth and loss parameters day 17*

$K_B = 0.0227$ cells $cell^{-1} h^{-1}$.

$I_A = 0.011$.

$I_M = 0.014$.

$K_L = 0.0114$ cells $cell^{-1} h^{-1}$.

The relative number of cells recognised as apoptotic is the apoptotic index.

Assuming that apoptosis is responsible for all the cell loss at day 11, then

$$N_{A'} = K_L \text{ at day 11.}$$

*Equation of Wyllie (1975) (i)*

$$\frac{N_M}{N_A} = \frac{n_M}{n_A} \cdot \frac{t_A(k+2r_A)}{t_M(k+2r_M)}$$

*Adaptation of Wyllie's equation (ii)*

$$\frac{K_{B'}}{K_L} = \frac{I_{M'}}{I_{A'}} \cdot \frac{t_A(k+2r_A)}{t_M(k+2r_M)}$$

$$N_M = K_{B'}$$

$$\frac{n_M}{n_A} = \frac{I_{M'}}{I_{A'}}$$

Thus, substituting in equation (ii),

$$\frac{0.021}{0.002} = \frac{0.014}{0.027} \cdot \frac{t_A(3+2[3])}{1.3(3+2[5])} = \frac{t_A 0.126}{0.456}$$

$$t_A = 38.1 \text{ h}$$

To find the average value of the rate of entry into apoptosis, $N_A$, restate equation (ii) to apply to the parameters of day 17 post implant as

$$\frac{K_B}{N_A}=\frac{I_M}{I_A}\cdot\frac{t_A(k+2r_A)}{t_M(k+2r_M)} \quad \ldots equation\ (iii)$$

substituting in equation (iii),

$$\frac{0.0227}{N_A}=\frac{0.014}{0.011}\cdot\frac{38.1(3+2[3])}{1.3(3+2[5])}$$

$$\frac{0.0227}{N_A}=\frac{4.8}{0.159}$$

$$N_A=0.00075\ \text{cells cell}^{-1}\text{h}^{-1}$$

This is thus the average cell loss rate due to apoptosis that is in operation 17 days post implant.

The overall cell loss rate on day 17 = 0.0114 cells $cell^{-1}$ $h^{-1}$.

Thus apoptosis on average accounts for 6.6% of the overall cell loss in this sarcoma.

The authors are most grateful for the advice and support, of Dr J. Denekamp and her team at the CRC Gray Laboratory, Mount Vernon Hospital, for the supply and implantation of the tumour bearing animals: the constructive advice of Dr A.H. Wyllie was also most gratefully accepted. We would also like to thank Mr D. Peksa at the Cardiff University Computer Centre for his help with the production of the computer derived graphics. Miss S. Jones' technical assistance and the photographic services of Mr V.T. Williams were greatly appreciated: the work was financially supported by SERC.

### References

AHERNE, W.A., CAMPLEJOHN, R.S. & WRIGHT, R.A. (1977). *An introduction to cell population kinetics.* Edward Arnold.

AHERNE, W.A. & CHALLONER, D. (1983). A reappraisal of the stathmokinetic technique: metaphase degeneration and its correction. *Virchows Arch. B (Cell Pathol.)*, **42**, 111.

BARKA, T. & ANDERSON, P.J. (1962). Histochemical methods for acid phosphatase using hexazonium pararosaniline as coupler. *J. Histochem. Cytochem.*, **10**, 714.

BELLAMY, D. & HINSULL, S.M. (1978). Density dependant mitosis in the Walker 256 carcinoma and the influence of host age on growth. *Eur. J. Cancer*, **14**, 747.

BELLAMY, D., HINSULL, S.M. & PHILLIPS, J.G. (1976). Factors controlling growth and age involution of the rat thymus. *Age Ageing*, **5**, 12.

BOWEN, I.D. (1984). Laboratory techniques for demonstrating cell death. In *Cell Ageing and Cell Death* (Eds. Davies, I. & Sigee, D.C.), p. 5. Cambridge University Press.

BOWEN, I.D. & LEWIS, G.H.J. (1980). Acid phosphatase activity and cell death in mouse thymus. *Histochem. J.*, **65**, 173.

BULLOUGH, W.S. (1967). *The Evolution of Differentiation*, p. 108, Academic Press, London and New York.

CARSWELL, E.A., OLD, L.J., KASSEL, R.L., GREEN, S., FIORE, N. & WILLIAMSON, B. (1975). An endotoxin induced serum factor that causes necrosis of tumours. *Proc. Nat. Acad. Sci. USA*, **72**, 3666.

COLUMBANO, A., LEDDA-COLUMBANO, G.M., RAJALAKSHMI, S. & RAO, P.M. (1984). The occurrence of cell death (apoptosis) in preneoplastic and neoplastic liver cells. A sequential study. *Amer. J. Pathol.*, **116**, 441.

DENEKAMP, J. (1984). Vascular endothelium as the vulnerable element in tumours. *Acta Radiol. Oncol.*, **23**, 217.

DUVALL, E. & WYLLIE, A.H. (1986). Death and the cell. *Immunol. Today.*, **7**, 115.

KERR, J.F.R., WYLLIE, A.H. & CURRIE, A.R. (1972). Apoptosis: a basic biological phenomenon, with wide ranging implications in tissue kinetics. *Br. J. Cancer*, **26**, 239.

LEWIS, G.H.J. & BOWEN, I.D. (1985). A methacrylate embedding technique for combined autoradiography and acid phosphatase histochemistry. *Histochem. J.*, **17**, 467.

LOEWENSTEIN & GALLILY, R. (1984). Studies on the mechanism of macrophage mediated tumour cell lysis induced by Mycoplasma orale. *Isr. J. Med. Sci.*, **20**, 895.

MENDELSOHN, M.L. (1960). Autoradiographic analysis of cell proliferation of spontaneous breast cancer of the CH3 mouse. II. Growth and survival of cells labelled with tritiated thymidine. *J. Natl Cancer Inst.*, **25**, 485.

NAG Fortran graphics supplement, *Numerical Allegraphics Routines on the Honeywell Multics computer system.* Routines EO2ACF and JO6EAF (copyright 1985).

PUCK, T.T. & STEFFEN, J. (1963). Life cycle analysis of mammalian cells. *Biophys. J.*, **3**, 379.

RUSSELL, J.H., MASAKOWSKI, V., RUCINSKY, T., PHILLIPS, G. (1982). Mechanisms of immune lysis. *J. Immunol.*, **128**, 2087.

SARRAF, C.E., JONES, S.L. & BOWEN, I.D. (1986). The diagnostic pathology of the effects of isotretinoin and tumour necrosis factor respectively on two murine sarcomas. *Proc. Roy. Microscop. Soc.*, **14**, S 60.

SEARLE, J., COLLINS, D.J., HARMAN, B. & KERR, J.F.R. (1973). The spontaneous occurrence of apoptosis in squamous carcinomas of the uterine cervix. *Pathology.* **5**, 163.

STEEL, G.G. (1977). *Growth Kinetics of Tumours.* Oxford University Press.

SYLVEN, B. & NIEMI, M. (1972). Histochemical evidence of cell death in transplanted tumours. *Virchows Arch. B. (Cell Pathol.)*, **10**, 127.

TANNOCK, I.F. (1968). The relationship between cell proliferation and the vascular system in a transplanted mouse mammary tumour. *Br. J. Cancer*, **22**, 258.

TANNOCK, I.F. (1970). Population kinetics of carcinoma cells, capillary endothelial cells and fibroblasts in a transplanted mouse mammary tumour. *Cancer Res.*, **30,** 2470.

WEAVER, J.A. (1955). Changes induced in the thymus and lymph nodes of the rat by administration of cortisone and sex hormones, and by other procedures. *J. Pathol. Bacteriol.*, **69,** 133.

WEINBERG, J.B. & HANEY, A.F. (1983). Killing by human blood monocytes and human peritoneal macrophages: lack of alteration by endotoxin or quenchers or reactive oxygen. *J. Natl Cancer Inst.*, **70,** 1005.

WRIGHT, N.A. & APPLETON, D.R. (1980). The metaphase arrest technique, a critical review. *Cell Tissue Kinet.*, **13,** 643.

WYLLIE, A.H. (1975). *Apoptosis in the rat adrenal cortex.* Ph.D thesis. University of Edinburgh, p. 149.

WYLLIE, A.H. (1980). Glucocorticoid-induced thymocyte apoptosis is associated with endogenous endonuclease activation. *Nature,* **284,** 555.

WYLLIE, A.H., MORRIS, R.G., SMITH, A.L. & DUNLOP, D. (1984). Chromatin cleavage in apoptosis: association with condensed chromatin morphology and dependence on macromolecular synthesis. *J. Pathol.*, **142,** 67.